# The Emotional Experience of Mexican Women with SARS-CoV-2 during Pregnancy―A Qualitative Study

**DOI:** 10.3390/healthcare11202785

**Published:** 2023-10-21

**Authors:** María Eugenia Gómez-López, Vania Aldrete-Cortez, Aline González-Carpinteiro, Rosa Mendizábal-Espinosa, Liliana Bobadilla

**Affiliations:** 1 Neuroscience Department, National Institute of Perinatology (INPer), Mexico City 11000, Mexico; 2 Laboratory of Neuroscience and Cognitive Development, School of Psychology, Universidad Panamericana, Mexico City 03920, Mexico; valdrete@up.edu.mx (V.A.-C.); 0209222@up.edu.mx (A.G.-C.); 3 Social Research Institute of London, University College London, London WC1E 6BT, UK; r.mendizabal@ucl.ac.uk; 4 Nacer Temprano, Vivir en Grande, Civil Association, Tlalnepantla de Baz 54080, Mexico; 5 Hospital of Gynecology and Obstetrics “Dr. Luis Castelazo Ayala”, Instituto Mexicano del Seguro Social, Mexico City 01090, Mexico; lbobadilla@up.edu.mx

**Keywords:** emotional distress, pregnancy, comorbidities, COVID-19, emotional support

## Abstract

Pregnant women have been considered a high-risk group for SARS-CoV-2 infection; the impact of the disease on the health of a mother and her child is still being studied. The emotional impact of the pandemic on pregnant women has been extensively studied. Emotional distress is proposed as a perspective to explain the emotional manifestations in women during this stage as something common rather than pathological. The objective of this study was to explore the emotional experience of women who tested positive for SARS-CoV-2 towards the end of their pregnancy, during the first and second waves of COVID-19 in Mexico. A qualitative study was carried out: 18 pregnant women with COVID-19 were interviewed. A thematic analysis of the data was performed, resulting in 3 main themes and 14 subthemes. The COVID-19-infected mothers-to-be experienced mild to moderate emotional distress. It was more intense for those with comorbidities. This distress was aggravated during obstetrical complications and comorbidities, as well as during COVID-19 and postpartum. The emotional distress was appeased by both the perception of medical care and social support. The emotional distress of pregnant women with COVID-19 requires emotional support to reduce its impact.

## 1. Introduction

After the official declaration of the COVID-19 pandemic and the subsequent health emergency in March 2020 [1], pregnant women and their babies were considered a vulnerable group [2] due to the uncertainty surrounding the effect of the disease on both maternal and foetal health. Furthermore, due to hormonal changes during gestation, women may be partially immunosuppressed and more vulnerable to respiratory infections, bypassing questions on the impact on the mental health of mothers-to-be [3,4,5]. In addition to the concern of the disease, to contain it, the World Health Organization established global measures to suppress contagion, such as lockdowns and social distancing, which had a significant impact on different aspects of the life of the population, particularly on mental health [6].

The emotional toll of the pandemic on pregnant women has been the subject of numerous studies that aimed to detect symptoms of anxiety [7,8], depression [9,10], stress [11], and post-traumatic stress disorder [12] through screening instruments. However, it is worth noting the caution expressed by Ahmad and Vismara [13] regarding the limitation of these instruments in distinguishing between transitory maternal discomfort, such as emotional distress, and more clinically significant psychopathological conditions. This distinction is crucial for gaining a comprehensive understanding of the emotional impact and for determining the most appropriate intervention strategies.

Emotional distress comprises a range of both physical and mental symptoms commonly linked to typical mood fluctuations experienced by most individuals [14]. While, in certain instances, it may signal the onset of more serious disorders, in others, it represents a transient emotional response that is contextually expected [15]. This conceptualisation has challenged the prevailing diagnostic classification system and the problem of labelling responses to various life circumstances as disorders [16], as well as their overdiagnosis [17].

In this regard, Velasco [18] suggests that women get sick more often due to sociocultural pressures, as well as social norms and expectations that increase their vulnerability to the health risks that they live with, which causes them mental distress; additionally, Burin [19] offers an alternative perspective on emotional distress in women, emphasizing its transient and context-dependent nature as a response to various life situations within a specific social context, moving away from a strictly psychopathological view. This broader perspective has also been applied to the study of emotional well-being in pregnant women, helping to elucidate the primary emotional experiences during this phase [19].

To date, limited research has explored the psychological repercussions of COVID-19 on women infected during pregnancy. These studies have revealed notable rates of moderate depression and anxiety [20], alongside concerns about mortality, the welfare of their infants, the effect of isolation, and societal stigmatisation [21].

In Mexico, some studies involving pregnant women have been conducted to assess the prevalence of depression, anxiety, and stress, revealing significant percentages of these conditions [22]. Furthermore, research has explored the role of emotional support through social media channels [23].

A number of previous research studies [24,25] underscore the importance of qualitative research into the effects of COVID-19 on mental health. Such studies are necessary for delving deeper into individuals’ emotional responses both prior to and during the pandemic, shedding light on their experiences, perceptions, fears, and the disruption that they faced.

Some studies have used this methodology, but none have specifically delved into the emotional impact of the disease on expectant mothers. Consequently, there remains a pressing need for further qualitative research within this population.

In the context of Mexico, perinatal mental health remains an unaddressed concern. Despite the existence of an official standard [26] emphasizing the necessity for psychological support within this demographic, psychological care for pregnant women has yet to be fully integrated into their comprehensive health care [19]. Therefore, this study aimed to explore the emotional experience of pregnant Mexican women who tested positive for SARS-CoV-2 during their last trimester based on two models: (a) the biopsychosocial health model by Velasco [18] and (b) the explanatory model of psychological distress in pregnant women with obstetrical complications [19]. The first posits that the concept of health and disease involves a dynamic interplay of biological and physiological factors within a social context and that it is intertwined with individuals’ subjective experiences and coping mechanisms. However, the second model suggests that emotional distress encompasses a range of transient emotional expressions that women may encounter during pregnancy, as part of their reproductive journey. The intensity and nature of this distress can vary based on the medical diagnosis of the complication and the unique pregnancy experience of the individual.

## 2. Materials and Methods

This qualitative study is part of a broader project. To collect the sample, a subgroup of women who had participated in the first stage of the project [27] was intentionally invited to participate. A qualitative analysis of the data was conducted using a thematic analysis proposed by Braun [28], following six steps: (1) familiarisation with the data; (2) the creation of a list with categories and preliminary codes; (3) the identification of the main topics; (4) the revision of potential topics; (5) the definition and naming of topics; and (6) the writing of the final report. 

### 2.1. Study Design

The design is a collective case study, as the study of multiple cases makes it possible to better illustrate the problems studied and to provide different perspectives [29], and, given the unprecedented situation that was being experienced due to the pandemic, this design gave us the possibility to address the phenomenon in a comprehensive manner. The sampling method was homogenous and intentional, and the data were obtained by means of semi-structured interviews based on an interview guide specifically created for this study (Table S1: Interview Guide). The invitation to participate was delivered via telephone to women whose babies were part of the group of mothers who tested positive for SARS-CoV-2 during the first stage of the project “Neurodesarrollo de neonatos de madres con y sin COVID-19” (“The neurodevelopment of neonates birthed by mothers with and without COVID-19”) published by Aldrete-Cortez et al. [27], to which this study belongs. When the obtained data reached saturation, the recruitment of participants stopped. The mothers recruited for this study tested positive for SARS-CoV-2 from 23 April 2020 to 8 February 2021, which corresponded to the first and second waves of the COVID-19 pandemic in Mexico; however, the interviews were carried out from 24 November 2020 to 12 August 2021. The interviews were recorded and lasted approximately an hour.

### 2.2. Study Participants and Setting

The interviews were conducted with women who tested positive for SARS-CoV-2 during the last trimester of their pregnancy. Overall, 18 women of age were interviewed. The inclusion criteria were as follows: participants had to (1) be older than 18 years; (2) be part of the project “The neurodevelopment of neonates born by mothers with and without COVID-19”; (3) have given birth to their babies during the first 2 waves of the pandemic (from 23 April 2020 to 8 February 2020); and (4) have a positive RT-PCR-SARS-CoV2 test during the last weeks of their pregnancy (≥31). Excluded from the study were those who had a neurological or psychiatric disorder that would have prevented them from answering the interview on their own, as well as those who did not accept to be recorded or who did not finish the interview.

The interviews were made by phone call with a set appointment and lasted approximately an hour; they were recorded and then transcribed by the researchers’ support group. No financial incentive was offered to the respondents for their participation in the study. The sociodemographic and medical information was obtained from hospital records. The age of the participants ranged from 18 to 39 (±5.39) years old. 

### 2.3. Data Analysis

This study was guided using the criteria established by the COREQ (Consolidated Criteria for Reporting Qualitative Research) [30].

The thematic analysis of the data was carried out as follows: (1) two of the researchers (M.E.G.L. and A.G.C.) read the interview transcripts to familiarise themselves with the data; (2) the main researcher (M.E.G.L.) created a list of categories and preliminary codes; (3) both researchers (M.E.G.L. and A.G.C.) identified the main themes stemming from the data; (4) both (M.E.G.L. and A.G.C.) also reviewed the list to reach a consensus; (5) the themes obtained were reviewed once more along with their relation to the data and the research questions (M.E.G.L. and A.G.C.); and (6) the final report was written (M.E.G.L., R.M., V.A.C., L.B., and A.G.C.).

### 2.4. Ethical Considerations

This study was approved by the hospital’s Research and Ethics Committee (R-2020-785-151, CIP-PI-054-2020-1, and E2003), and it was developed in accordance with the ethical principles of research. 

An informed consent form was sent electronically to the participants—via WhatsApp—before the study, and their authorisation to record the interviews was requested; likewise, their participation and the purpose and content of the study were thoroughly explained to them.

## 3. Results

The average age of the participants was 28.9 years old (±5.3); most of them had a partner (90%); the majority (90%) had ≥12 years of schooling; around 44% were housewives; and almost half of them (49.3%) were experiencing their first pregnancy. Additionally, 63.6% of the participants had obstetrical complications or comorbidities, and among the most frequent were the threat of miscarriage and/or preterm delivery, gestational diabetes, and preeclampsia; however, the most frequent health antecedents were overweight or obesity and metabolic, cardiac, and hepatic diseases. Similarly, 67% of the women interviewed stated that their pregnancy was unexpected, while 27.7% reported having lost a family member to COVID-19. Furthermore, all the mothers presented mild to moderate symptoms consistent with COVID-19, according to Gandhi et al. [31]. None of them needed intensive care or mechanical ventilation. All women were discharged home without complications.

To analyse the data according to the obtained information, the sample was divided into two groups: (1) women with comorbidities/with obstetric complications (wwC/wOC) (11 participants) and (2) women without comorbidities/without obstetric complications (wwoC/woOC) (7 participants). The sample was divided in this way with the intention of finding differences in the manifestation of emotional distress, both because of the theoretical model that we were working with and to observe how the comorbidities related to SARS-CoV-2 behaved in pregnant women and whether this had an impact on their emotional health.

Table 1 shows the main characteristics of the sample.

Three main themes and fourteen subthemes emerged from the data analysis, all of which are shown in Table 2.

### 3.1. Getting Pregnant during the Pandemic

Experiencing their pregnancy during the pandemic had a significant emotional impact on the participants because of what the disease could mean for their health and that of their babies.

#### 3.1.1. Perception of Health during Pregnancy: Care and Complications

Two-thirds of the respondents (67%) did not plan their pregnancy, which provoked emotional reactions ranging from joy and worry to surprise, fear, tension, confusion, and uncertainty. 

Additionally, these 11 participants were also concerned about their own comorbidities and obstetric complications.

*“(...) during my pregnancy I found out that I had hypothyroidism. Soon after I got pregnant, I was diagnosed with high blood pressure. To be honest, I was very controlled–obviously–in case I got preeclampsia. It was a complicated pregnancy indeed”*

(P11).

Nevertheless, the participants followed the care instructions given to them by their doctors, including going to their medical appointments, eating healthy, drinking more water, exercising, not exerting themselves, and not going outside.

#### 3.1.2. Perception of Medical Care during Pregnancy and Its Resolution

The unprecedented situation caused by the global pandemic and confinement represented a change in pregnancy care measures and access to medical care, as well as insufficient information about the impact that the disease could have on mothers-to-be and their babies. This created a context of uncertainty in which the participants experienced their pregnancy.

The search for medical care was a concern for the interviewees from the beginning of their pregnancy because having a monthly follow-up was a priority but so was attending a hospital that would guarantee their care during labour. This led many of them to seek dual medical care: private (with a private doctor who they knew and trusted) and public (a hospital that could treat them during the resolution of their pregnancy). However, several women expressed fear of going to the hospital or clinic for fear of contracting SARS-CoV-2: “(...) at the beginning I kept going (to my appointments) obviously afraid of getting infected there, but going itself was already stressful enough, because you didn’t know if there were any (appointments) or not, or what is going on...” (P10).

Concerns about the economic situation resulting from the health emergency also played a role in their decision to seek care in a public hospital.

All participants received several follow-up visits during their pregnancy, and once they started care at the tertiary hospital specialising in obstetrics and gynaecology where this study was conducted, none of them had problems accessing said care despite the circumstances: “I went to my appointments every month; well, when I started seeing the gyno they were weekly, and there were several—around 10 appointments, I think” (P8). 

Most of the interviewees also mentioned that the medical care was good, that they were given the necessary tests for their condition, and that they were provided with the medicines that they required. However, some expressed that the change in hospital care protocols due to the pandemic had also led to confusion and misunderstandings with the health personnel.

#### 3.1.3. Social Support: Environment and Pandemic

For the participants, living their pregnancy in confinement meant changing their daily routine; being locked up and, in some cases, away from their families; working at home or having to go out to work at the risk of infection; having to support their children with their remote classes; putting aside their plans; worrying about the economy and unemployment; or even being separated from their partner. However, at the same time, most agree that the confinement allowed them to simultaneously enjoy their pregnancy more.

*“I felt calm and happy–the pandemic didn’t affect me much. I was at home because I was taking care of myself. It did affect me socially ‘cause people wanted to visit me but they couldn’t”*

(P9).

The confinement allowed them to strengthen their relationship with their children and their partner, but they also sought support from family and friends, both during their pregnancy and later on. These circumstances meant a time of adjustment for the members of their families, who had to assimilate to the arrival of a new member along with the adaptation to the changes caused by the confinement.

#### 3.1.4. Prenatal Emotional Distress

The participants went through their pregnancy with a lot of worry, fear, anxiety, and tension. These emotions revolved around two main themes: (1) their health and that of their baby and (2) the impact of COVID-19. Nevertheless, a significant difference was found in the emotional alterations shown by women who had comorbidities (group wwC/wOC) and those who did not (group wwoC/woOC), because the former lived their pregnancy worried not only about the pandemic but also about their health status and possible complications.

Regarding their health and that of their baby, group wwC/wOC reported the following emotional manifestations: concern about medical care; anxiety about feeling the baby move; isolation; fear of their own death, that of their baby, or both; fear for the baby’s health; fear of losing the baby; concern about being pregnant with comorbidities; fear and shock about complications and nervousness due to the actual labour; fear of pain; pent-up emotions; crying; anger; frustration; helplessness; sadness; anguish; decay; discomfort; sadness for not being able to play with their older children; and weight gain due to stress.

As for the impact of COVID-19, this same group presented tension due to unemployment, insomnia, sadness due to the loss of a family member, suffocation, nervous breakdowns, pressure, depression, a fear of falling ill, a fear of leaving their children alone, financial worries, despair, and anxiety.

*“I was afraid and anxious because of money. I was nervous about labour because it was my first baby, I was more nervous about that than about the disease (COVID-19)”*

(P6).

However, the main emotional manifestations of group wwoC/woOC were less intense and more related to the impact of COVID-19, such as financial worries, sadness about contracting COVID-19, concern for the health of their children and their pregnancy, and depression due to being separated from their partner.

### 3.2. Getting Infected with SARS-CoV-2 before Giving Birth

Contracting COVID-19 during the last weeks of their pregnancy had a great emotional impact on the participants due to the risks that it posed to them and their babies, as well as being separated from their newly born children and kept in isolation at the hospital.

#### 3.2.1. COVID-19 Information and Care Measures

The participants already had some general information about the disease, the symptoms, and the risks of catching it. They perceived it as dangerous not only because people could die but also because it was unknown and new, with little information about its effect on pregnant women. It creates fear and panic, as it affects people with chronic diseases more, has long-term side effects, and is rapidly spread. This perception contributed to increasing their emotional discomfort; however, some also perceived it as a flu or a myth, and they felt it was better not to be informed or pay attention to alarmist news to avoid getting upset.

*“I knew COVID-19 was dangerous, that’s why I worried, because they said it was really dangerous; whenever I knew of someone who had got Covid, it was because they had died”*

(P17).

Likewise, the participants knew the preventive measures to protect themselves from the disease, such as using masks, washing their hands, changing their clothes, using hand sanitisers and face shields, not going outdoors, and disinfecting.

#### 3.2.2. Infected Mother

The participants tested positive for SARS-CoV-2 between weeks 31 and 39 of their pregnancy. They all assumed that their infection was caused by either their partner or a close relative. The most commonly reported symptoms of COVID-19 were sleepiness, vomiting, diarrhoea, stomach aches, sore throat, a lack of smell and taste, fatigue, a dry and irritated throat, chills, back pain, cough, breathing difficulties, flu-like symptoms, no feeling of foetal movements, headaches, fever, suffocation, and chest pain; there was one asymptomatic case.

*“That Thursday I decided to visit the doctor because I was feeling really bad. I was quite thirsty and nothing quenched it. I had the chills too. I was already feeling too weak, I couldn’t get out of bed, and when I got to the hospital and said I had those symptoms, I was taken to a restricted area…”*

(P9).

#### 3.2.3. Resolution of a Pregnancy with COVID-19

Giving birth while being infected with COVID-19 was quite a difficult situation for the participants, filled with uncertainty and fear of complications. Out of the participants in both groups, 66.6% had a C-section, and the rest opted for a natural birth. 

In this context, the women in group wwC/wOC suffered from complications during the resolution of their pregnancy, such as a premature rupture of membranes, pre-eclampsia, and a low-lying placenta. As for the babies, the most common complication was oligohydramnios, and only one baby had temporary breathing problems that required oxygen (via the use of prongs).

*“Yes, I had Covid. I no longer felt my baby moving so I left for the hospital and I was told I no longer had any liquid in me, so they told me I needed an urgent C-section”*

(P15).

In contrast, the women from group wwoC/woOC had no complications; nevertheless, like in the previous group, some of their babies had oligohydramnios.

Regarding the perception of medical care, some participants described it as good and attentive, with good care from the staff; others, however, stated that, in some cases, it was slow due to a lack of staff, with bad treatment and cancelled appointments, little information given to their relatives during hospitalisation, little communication with the family, a lack of indications for post-COVID-19 care, and a lack of a discharge protocol for both patients and babies after the disease.

#### 3.2.4. Isolation and Recovery from the Disease

The participants’ recovery after COVID-19 involved being in isolation during their hospital stay and afterwards, which meant that they had to be separated from their babies and family members for a few days up to four weeks. Those who were hospitalised mentioned that what affected them the most was being separated from their babies, not receiving information about them, and not being able to be in touch with their families. “I met my boy almost a month after because I was hospitalised for 14 days, and they told me I had to be in isolation for another 15 days” (P1).

Others mentioned that they decided to isolate themselves as a family or that it was hard for them because they had to look after their older children.

All participants perceived different signs of recovery from the disease, such as being moved to a non-COVID-19 area in the hospital; being able to get up, go to the toilet, and fend for themselves; communicating with their family; having an appetite, tasting food, and sensing smells again; no longer being contagious; being able to breathe well; testing negative for SARS-CoV-2; and being discharged from the hospital.

They all also mentioned having been left with aftereffects of the disease that they had to deal with for some time, such as joint pain, fatigue, shaky legs, coughing, a lack of smell and taste, hives, memory problems, suffocation, back pain, weak lungs, headache, body aches, secretions, hair loss, a bitter taste in food, and a decompensation of blood pressure.

The post-COVID-19 care that followed was isolation from their family; cleaning and disinfecting with alcohol; changing clothes and bathing; using masks, hand sanitisers, and face shields; putting themselves in quarantine; washing their hands; taking vitamins; going somewhere else; and only approaching the baby for feeding.

#### 3.2.5. Impact of the Disease on the Family and Losses

The emotional experience of the participants when facing the disease was also nuanced by its impact on their families and the losses that it caused. All participants reported that one or more members of their family were ill, some mildly and some severely, and several suffered one or more losses. The grief experienced in this situation was characterised by disbelief; crying; sadness; depression; pain; worry; stress; the need to vent; resignation; acceptance; pain because the deceased family members were unable to meet the baby; thinking that they no longer suffered; not being able to experience grief well; grieving in solitude; the grief in itself being a heavy blow, a difficult situation, and a traumatic event; difficulty in assimilating to the loss; stopping their grieving to care for the baby; distracting themselves with the baby; and the fear of getting sick and infecting their children.

*“My mum got infected and they both passed away, my dad on May 3 and her on May 7. But I didn’t know because I was hospitalised. I still had hopes for my mum to resist and get to know my son but it didn’t happen. A month later, my sister dies, and I told myself: ‘We can’t be living this situation’; for me it was more like a dream or a nightmare”*

(P3).

#### 3.2.6. Emotional Distress Due to COVID-19 and Life Changes

The emotional experience of the participants with COVID-19 during their pregnancy was primarily focused on worrying about the baby and their family, and it was more intense for the women in group wwC/wOC. This group experienced the disease with tension, crying crises, anxiety, memory problems, despair, anguish, fear, depersonalisation, uncertainty, doubt, a lack of acceptance, worry, avoidance, grief, anger, rage, irritability, sadness, loneliness, instability, shock, trembling, surprise, tension, difficulty in managing emotions, a feeling of losing control, feeling “contaminated”, fear of getting worse, fear of falling asleep under anaesthesia, fear of affecting the baby, fear of the baby forgetting them because of the separation, suffering from not being able to be with the baby, worrying about the birth, and fear of not seeing their family again.

*“I was so struck by it… from the moment I was put inside that capsule [during the birth of my baby] I said, ‘Oh my God, what is going on?’—I felt contaminated. I was afraid because the doctors were using their suits; it was something really weird and so different from my other deliveries”*

(P8).

However, the emotional alterations of the participants in group wwoC/woOC were less intense and were characterised by a concern for the health of the baby and that of their family, sorrow, poor appetite, sleepiness, solitude, sadness caused by the separation from their baby, the fear of getting them infected, a fear of the surgery, and a fear of dying. 

The perception of the participants on the life changes that they went through as a result of the disease can be split into negative and positive perceptions. In terms of the negative ones, they expressed living with the fear of getting infected again, not having physical contact with others, feeling uneasy about having to go out, a fear of touching the baby, fear, shock, and trauma. As for the positive changes, they expressed being more cautious, feeding themselves better, following the care and hygiene measures, giving affection, being more protective, feeling grateful for having overcome the disease, feeling calmer, being more patient, appreciating what they had more, and having a more united family.

### 3.3. Coming Back Home after the Delivery and COVID-19

Leaving the hospital and returning home after giving birth and recovering from COVID-19 had a major emotional impact on the participants because some had to return without their baby, as they had to leave them in the care of a relative to avoid infecting them, and others could only see them in photos or to feed them because they continued in voluntary isolation.

*“As I couldn’t have him (the baby) with me, they took him away because I had Covid. A cousin of mine took care of him for me. She took the baby and took care of him while I recovered. It was more or less for about 15 days, and that was when the sadness increased”*

(P10).

#### 3.3.1. Health, Complications, and Postpartum Recovery

The health of the interviewees after the resolution of their pregnancy was characterised by some complications, such as uncontrolled blood pressure and gastric problems (colitis, gastritis, and constipation), but the greatest impact was observed in their emotional state, as they experienced changes in their mood (sadness, anxiety, despair, and worry) related to the care of the baby and their other children, as well as to the family adjustments made around the new member, while others experienced changes in their mood associated with the confinement and the fear of getting infected. Overall, they said that they recovered quickly and with no special care.

*“I felt so sad, I wanted to cry over everything. I’m a cry baby, and as I was kind of depressed, I cried so much more. I felt sad, disappointed, nervous, worried, and felt really bad about it later. I didn’t even want my mum to leave me when I recovered from giving birth”*

(P17).

#### 3.3.2. The Search for Medical Care

Those with complications such as uncontrolled high blood pressure had to consult a specialist for follow-up care, which was difficult to obtain because of the pandemic. Others preferred to continue without any medical follow-ups for fear of going out to a medical appointment or having the lack of time to do so; however, they all focused on getting paediatric care for their babies.

*“I was checked and treated at the doctor’s. I was told I didn’t need to stay, it was just the postpartum. I was also going to the clinic for that, for a follow-up, but they always told me there were no doctors available”*

(P5).

#### 3.3.3. Family and Social Support

During postpartum, the participants had family support, mainly from their partner, but also from their parents, siblings, aunts, uncles, and cousins, as well as their in-laws, specifically their mother-in-law, who supported them in terms of house chores, caring for their other children, feeding them, and doing all that was needed for them to be alright. In some cases, their relatives lived in the same house; in others, they visited them despite the risk. “My sister, an aunt and my cousins supported me; and they also did so when my father got seriously ill for my brother had to be with him. And so did my mother-in-law” (P11).

Family support was also important during postpartum for emotional and instrumental support. Social media also played a relevant role in being a source of support in terms of buying products, and it also became a place for lonely mothers who were going through bad times to network.

*“A Facebook group I’m in was of great help. I shared what I was going through with them and the mums did some incredible work and supported us a lot. They sent us lots of things so we could remain isolated”*

(P2).

#### 3.3.4. Emotional Distress during Postpartum and Life Changes

The participants’ adjustment to the changes caused by motherhood, as well as those related to the pandemic, the disease, the care and recovery, and the grief from the loss of family members, generated a range of emotions mainly related to the following two themes: (1) The baby: life changes caused by COVID-19; sadness for being separated from their baby; despair caused by the COVID-19 preventive measures (masks); fear of infecting the baby, of not being able to touch them, of taking care of them, of not being able to breastfeed them; guilt; anguish for having to go out, for leaving the baby to go back to work; tension due to the increase in house chores; and economic anguish. (2) Delayed grieving: contained emotions, crying, isolation, anger, incredulity, frustration, impotence, despondency, stress, mood changes, worry, uncertainty, solitude, fatigue, anxiety, and sadness.

*“I felt somewhat despondent because I could not be in touch with my baby in the same way, we couldn’t go out—it was really stressful being like that. I think my mood changes were more frequent. The truth is I got mad about everything”*

(P15).

The life changes perceived due to both COVID-19 and the arrival of the new family member mostly implied a resignification of the lived experience: valuing more what one has; enjoying life, family, and their baby more; taking better care of oneself; taking into account that life can change from one moment to another; and assuming more responsibilities. However, the participants continued to be afraid of the disease, of becoming infected, and of dying, and they considered that having become ill with COVID-19 was a bad experience.

## 4. Discussion

This study explored the emotional experience of women who tested positive for SARS-CoV-2 in the last trimester of their pregnancy, during the first and second waves of the COVID-19 pandemic in Mexico. Most participants experienced mild to moderate emotional distress; however, the group of women who suffered from comorbidities was the most affected. We identified three moments of crisis that caused increased distress: (1) the occurrence of complications and comorbidities of the pregnancy; (2) the time of confirmed infection with COVID-19; and (3) the postpartum period. However, the medical care and the social support that this group of women received were factors that contributed to reducing their emotional distress.

It is important to consider the context in which this study was carried out (during the first and second waves of the pandemic in Mexico). Up to that moment, little was known about the impact of COVID-19 on pregnant women and their babies, and neither vaccines for protection against contagion nor medication to reduce the risks of infection had been developed. The population was in lockdown for about three months, and economic activities were gradually reinitiated.

This study contributes to identifying the emotional condition that the pregnant population went through during the course of the disease and the pandemic; it also allowed us to understand the impact that the separation from their babies and their isolation in hospitals had on these women, which eventually resulted in changes in their mothering. This work highlights the need to generate medical care models during health emergencies that provide psychological support as part of obstetrical care under these circumstances.

The mild-to-moderate emotional distress experienced by the women who became ill with COVID-19 during their pregnancy in this study is consistent with that proposed by Ng [32], who suggested that the characteristics of emotional manifestations during the pandemic were reactive and responded to short-term adjustment and long-term adaptation problems and that, due to psychosocial factors, such as the uncertain future, the fear of contagion, confinement, life changes, and economic concerns, these manifestations were expected and, therefore, should not be psycho-pathologised.

It should be noted that, in this study, 63.6% of the interviewees had obstetrical complications (threatened miscarriage and/or preterm birth, gestational diabetes, and pre-eclampsia) or comorbidities (overweight or obesity and metabolic, cardiac, and hepatic diseases). This coincides with that proposed by the explanatory model of emotional distress in pregnant women [19].

Although it is known that pregnancy involves physical, psychological, and social changes that must be assimilated by women in order to adapt to motherhood [33], when it is experienced with complications and comorbidities, its psychological impact is greater [19].

We suggest that three moments of crisis exacerbated emotional distress: (1) the period of pregnancy; (2) the period of COVID-19 infection; and (3) the period of postpartum. During pregnancy, the fact that most of the participants had obstetric complications and/or comorbidities influenced their experience of emotional distress to a greater extent, mainly due to two types of factors: (1) those inherent to pregnancy and (2) those related to the pandemic. In terms of the first, the women lived their pregnancy with a lot of worry, tension, and fear. Similarly, the lack of pregnancy planning contributed to exacerbating this distress, which is consistent with the explanatory model mentioned above [19]. As for the factors related to the pandemic, it was identified that fearing for the health of the baby, the fear of getting infected with SARS-CoV-2, and economic concerns also contributed to increasing the emotional distress of the participants during their pregnancy in the same way that it is highlighted in other studies [34,35]. However, this finding is contrary to that of other studies that found no association between gestational complications and the emotional well-being of women [36].

In the second time of crisis, it was observed that the emotional manifestations of the participants became more acute when dealing with COVID-19 symptoms, especially in those with previous medical problems, with a predominance of fear for their health and for that of their baby. However, the greatest impact was related to being separated from the baby and isolated in the hospital, which is consistent with similar studies [37].

In contrast, despite the emotional impact caused by living their pregnancy during the pandemic and having COVID-19, the women valued the life changes brought about by the pandemic, focusing more on caring for their health and for that of their family, feeling grateful for having overcome the disease, spending more time with their baby and their other children, and enjoying family moments, among others, which is consistent with previous findings [38].

As for the last time of crisis—postpartum—we found that the women continued to fear infecting the baby, and some also decided to continue their isolation at home, which prevented them from caring for their child for several more days; this is similar to what was reported by Freitas-Jesus [21]. Similarly, several participants were left with sequelae from the infection, which also contributed to their isolation. In addition, most of the women reported that they had to make changes in the care of their baby, including taking extreme hygienic measures, such as the use of masks to approach and interact with the baby, which made them feel desperate and limited them, as reported in other studies [28]. It is possible that this influenced and changed the mothering of the participants, as affirmed by Chivers [34].

Over one-quarter of the participants experienced the loss of one or more family members to COVID-19. As a result, the women tended to postpone their grieving process to focus on their pregnancy and health, but it was not until the postpartum period that they allowed themselves to experience their grief, which led to prolonged emotional distress. This is consistent with other findings [38]; in addition, the characteristics of the pandemic meant that the women had to deal with different types of loss, as noted by Kumar [39].

Another possible form of grief is related to having lost the opportunity to experience the medical care required during their pregnancy and the difference between how they had hoped it to be and the actual experience due to the changes made in medical care to avoid contagion [38]; however, this needs to be further explored in future studies.

As a final part of this discussion, there are two important aspects that nuanced and contributed to mitigating the emotional distress of the interviewees: (1) their perception of the medical care and (2) the social support that they received.

It is important to note that the Mexican health authorities published guidelines with changes in obstetric care [40], which guided hospital care during the period of this study. These modifications had some influence on the medical care received by the participants; experiences from other countries indicate that the medical care of pregnant women was limited and affected, which caused increased anxiety [41]. In contrast, the participants in this study described their medical care as good and fast, with no restriction on medical visits and with all necessary facilities available, even if the priority was to deal with the health emergency caused by the pandemic while having a significant lack of beds and medical staff—all of which coincides with previous studies [42].

Several interviewees mentioned that there were no clear protocols for the care of their babies after COVID-19, which caused them confusion. They also faced difficulties when getting in touch with their relatives or when not being able to receive any visits during their hospitalisation, which, in turn, influenced their perception of the care and their emotional distress. This coincides with previous studies [43] stating that medical staff and maternity services were working under pressure, sometimes understaffed, which often made organisation and communication with relatives difficult.

As for perceived social support, in contrast to other countries where restrictions due to lockdowns had a strong impact on the support received by women during their pregnancy from family members and close friends [44], we found that the perception of social support received by the participants during the three times of crisis had an influence on mitigating the intensity of their emotional distress, despite the isolation and limited contact that they had with their family members during hospitalisation, which initially exacerbated their distress. 

In this regard, it is important to consider that around 23% of families in Mexico [45] are extended families (i.e., the families of origin and those of procreation live or spend a lot of time together); in addition, many Mexican families joined households during the pandemic to solve economic problems and to care for their children and elderly. In this sense, the extended family became an important support network during confinement [46].

Moreover, it should be noted that, in Mexico, only 17.5% of people remained in absolute confinement during the health emergency, while the rest remained in partial confinement—they could go out for shopping or medical consultations [47]. This could also explain why most of the interviewees had the support of their relatives and other close people during their pregnancy and postpartum since, in many cases, they lived in the same house or went to help them. This support allowed them to feel accompanied and helped in caring for their baby, which prevented emotional distress from flaring up, as noted in other studies [48].

The experience of the pandemic highlighted the need for health workers to be prepared to provide emotional support and accompaniment to patients during hospitalisation and isolation. Therefore, telemedicine and the use of technology to provide care were efficient alternatives in several countries [49]; our study highlights the need to implement their use in the Mexican health system.

In terms of psychological care for pregnant women, it is important to develop alternative online peer support groups, which could be led and supervised by psychologists or experts in perinatal education. This could alleviate the burden that medical personnel experience, considering the overload and pressure that they faced during the pandemic, while also generating new spaces that give women the confidence to work on their emotions, as proposed by Lega [50].

Despite the diversity of studies carried out on the impact of the pandemic on the mental health of pregnant women, there have not been many studies on this subject in Mexico; therefore, a qualitative study about the experiences of women with COVID-19 during pregnancy is an opportunity to delve deeper into the subjective experience of this population. Having conducted this study within one of the largest public medical institutions in the country, a broader picture of how medical care was experienced and how the health emergency was dealt with has been provided, and this might give us an approximation of the experience in the rest of the country.

As for limitations, this work was carried out with women who were entitled to social security, which guaranteed them medical care; therefore, women without access to social security should have been included in order to understand the emotional impact on a wider part of the population. 

Another limitation was that the interviews were conducted by telephone, which prevented us from observing the non-verbal communication of the participants, as well as their reactions. Similarly, some of these interviews had to be conducted despite the lack of a relaxed and distractor-free environment because the women sometimes did the interviews with their babies next to them and/or near other people who distracted them. Finally, the variation in the time that elapsed between giving birth and the interview may have influenced the participants’ recall and perception of the event and its emotional impact.

It is up to future research to study the grief experienced by pregnant women due to COVID-19 in depth.

## 5. Conclusions

The emotional experience of women who tested positive for SARS-CoV-2 during pregnancy is divided into three times of crisis where emotional distress is manifested, exacerbated, and prolonged.

During pregnancy, emotional distress is mild to moderate, and its intensity is heightened in women with comorbidities and/or obstetric complications and when the pregnancy was unplanned. Its impact on women is greater when related to the separation from their baby and their isolation while being hospitalised. 

The women’s positive perception of the medical care and the social support that they received contributed to mitigating emotional distress, and confinement was seen as an opportunity for them to spend more time with their families.

## Figures and Tables

**Table 1:** Characteristics of the participants.

Characteristics	Group wwC/wOC (11 Participants) *n* (%)	Group wwoC/woOC (7 Participants) *n* (%)
Age		
range	22–39 ± 4.52 *	18–29 ± 4.05 *
mean	31.5	24.8
Marital status		
with partner	9 (81.7)	7 (100)
without partner	2 (18.2)	0
Education level		
≤junior high school	2 (18.2)	0
≥senior high school	9 (81.8)	7 (100.0)
Occupation		
housewife	5 (45.4)	3 (42.8)
employee	3 (27.3)	3 (42.8)
trader	1 (9.1)	0
technical	1 (9.1)	1 (14.3)
professional	1 (9.1)	0
Pregnancies		
1	3 (27.3)	5 (71.4)
≥2	8 (72.7)	2 (28.6)
Pregnancy planning		
Yes	4 (36.3)	2 (28.5)
No	7 (63.6)	5 (71.4)
Type of pregnancy resolution		
delivery	3 (27.3)	3 (42.8)
C-section	8 (72.7)	4 (57.1)
Loss of relatives due to COVID-19		
Yes	2 (18.2)	3 (42.8)
No	9 (90.8)	4 (57.1)
Comorbidities		
cardiac	2 (18.2)	N/A
metabolic	2 (18.2)	N/A
advanced maternal age	3 (27.3)	N/A
overweight/obesity	5 (45.4) **	4 (57.1)
other	4 (36.3)	N/A
Obstetrical complications		
premature rupture of membranes	3 (27.3)	N/A
preeclampsia	2 (18.2)	N/A
gestational diabetes	4 (36.3)	N/A
other	2 (18.2)	N/A

Initially, 19 participants agreed to be interviewed; 18 of them completed the interview. wwC/wOC: women with comorbidities/obstetrical complications. wwoC/woOC: women without comorbidities/obstetrical complications. * DE ** participants with more than one comorbidity; N/A: not applicable.

**Table 2:** Themes and subthemes.

Main Themes	Subthemes
1. Getting pregnant during the pandemic	(a) Perception of health during pregnancy: care and complications
	(b) Perception of medical care during pregnancy and its resolution
	(c) Social support: environment and pandemic
	(d) Prenatal emotional distress
2. Getting infected with SARS-CoV-2 before giving birth	(a) COVID-19 information and care measures
	(b) Infected mother
	(c) Resolution of a pregnancy with COVID-19
	(d) Isolation and recovery from the disease
	(e) Impact of the disease on the family and losses
	(f) Emotional distress due to COVID-19 and life changes
3. Coming back home after the delivery and COVID-19	(a) Health, complications, and postpartum recovery (b) The search for medical care
	(c) Family and social support
	(d) Emotional distress due to COVID-19 and life changes

## Data Availability

The data obtained are not available because they are clinical cases from the hospital where this study was carried out, so anonymity and confidentiality must be preserved.

## Supplementary Materials

The following supporting information can be downloaded at: https://www.mdpi.com/article/10.3390/healthcare11202785/s1, Table S1: Interview Guide.
