# Supplementary material for: The Emotional Experience of Mexican Women with SARS-CoV-2 during Pregnancy―A Qualitative Study
Source: Healthcare (Basel). 2023 Oct 21;11(20):2785. doi: 10.3390/healthcare11202785 (PMC10606424; doi:10.3390/healthcare11202785)
Supplement: Supplementary file 1 [file healthcare-11-02785-s001.zip › healthcare-2615416-supplementary.pdf]

Table S1. Interview Guide

Themes	Questions	Matters to explore
1. Maternal health before pregnancy	How was your health before getting pregnant?	• Overall health<br>• Healthcare<br>• Eating habits, exercise, previous consumption of cigarettes, alcohol, and/or other substances; emotional state before pregnancy
2. Pregnancy before COVID-19	How were you experiencing your pregnancy before the disease?	• Symptoms and pregnancy planning; reaction of the woman, her partner and her family to the pregnancy<br>• Diagnosis of obstetrical complications; medical care<br>• Mood<br>• Relatives sick with COVID-19; relatives who died because of the disease<br>• Expectations towards pregnancy; changes experienced
3. Diagnosis of COVID-19	What was your experience like when getting sick with COVID-19?	• Number of weeks pregnant when getting infected<br>• Information about the disease<br>• Symptoms and diagnosis of the disease<br>• Treatment<br>• Experiences and emotions towards the disease<br>• Isolation and social support<br>• Medical care<br>• Experience of the disease in relatives<br>• Experience of quarantine
4. Pregnancy after COVID-19	How did you experience your pregnancy after the disease?	• Special care<br>• Recovery from COVID-19<br>• Mood<br>• Support received
5. Pregnancy resolution	How did you experience the resolution of your pregnancy?	• Type of pregnancy resolution<br>• Experience during pregnancy resolution<br>• Perception of medical care<br>• Complications<br>• Emotions during pregnancy resolution; mood<br>• Support from family and staff<br>• Hospital stay: experience and care<br>• Recovery<br>• Communication and information on the baby<br>• Hospital discharge
6. Postpartum	What was your postpartum experience?	• Going back home<br>• Care for the baby

	• Extension of isolation<br>• Support from family and others<br>• Medical care<br>• Special care<br>• Mood<br>• Life changes caused by COVID-19 and the new baby
